# Comment on “C-reactive protein to lymphocyte ratio combined with clinical features to construct a predictive model for upper gastrointestinal bleeding due to peptic ulcer”

**DOI:** 10.1016/j.clinsp.2025.100749

**Published:** 2025-09-20

**Authors:** Parth Aphale, Shashank Dokania, Himanshu Shekhar

**Affiliations:** Dr. D.Y. Patil Vidyapeeth (Deemed to be University), Pimpri, Pune, Maharashtra, India

*Dear Editor,*

We commend Song et al. for their timely and clinically meaningful contribution investigating the role of the C-reactive protein to Lymphocyte Ratio (CLR) in developing a predictive model for Upper Gastrointestinal Bleeding (UGIB) secondary to Peptic Ulcer (PU).^1^ With UGIB representing a life-threatening complication of PU, early risk stratification remains a key priority in gastroenterological care. This study is noteworthy for attempting to combine inflammatory biomarkers and clinical features into a practical, statistically validated nomogram for early risk prediction.

The authors demonstrated that CLR, alongside Helicobacter Pylori (HP) infection, ulcer stage, Nonsteroidal Anti-Inflammatory Drug (NSAID) usage, and Neutrophil-to-Lymphocyte Ratio (NLR), independently predict UGIB, with the resulting model yielding an AUC of 0.921. This level of discrimination is promising, especially considering that conventional risk scoring systems like the Rockall or Blatchford scores primarily focus on post-endoscopy or hemodynamic parameters, limiting their utility in early, pre-endoscopic triage. Importantly, the inclusion of CLR as a composite biomarker reflecting both inflammation (CRP) and immunocompetence (lymphocytes) deserves emphasis. Several recent studies have recognized CLR's prognostic significance in other gastrointestinal and systemic inflammatory diseases, underscoring its versatility as a dynamic risk marker.^2^^,^^3^ Its integration into a predictive model may thus add more nuance than single-parameter metrics.

However, there are notable considerations. First, while the internal validation is methodologically sound, the study's single-centre, modest sample size (*n* = 146) limits external generalizability. CLR values may vary across populations due to underlying infectious burdens or demographic differences. As seen in multicentre analyses of CLR in other GI diseases, calibration across institutions is necessary before broad clinical adoption.^4^ Second, the selection of PCT (Procalcitonin) as a variable, despite its exclusion as an independent risk factor, may reflect the confounding impact of acute infection. Future studies could explore whether combining CLR with serum gastrin or haemoglobin levels enhances predictive accuracy in distinguishing bleeding severity versus inflammation alone.^5^ Lastly, while the model has clear clinical potential, its translation into bedside or emergency department workflows will depend on tool accessibility. An app-based or EHR-integrated scoring system could help streamline implementation, especially in low-resource settings where pre-endoscopy decisions are often time-critical.

In summary, Song et al. offer a valuable framework for pre-emptive risk identification in PU-related UGIB. With further multicentric validation, CLR-enhanced models may help move clinical care upstream, enabling earlier, targeted interventions and reducing morbidity from late-presenting haemorrhage.

## Ethical approval

Not applicable.

## Funding

No funding was received for this research.

## CRediT authorship contribution statement

**Parth Aphale:** Methodology, Formal analysis, Visualization, Writing – original draft. **Shashank Dokania:** Data curation, Investigation, Writing – original draft. **Himanshu Shekhar:** Project administration, Writing – review & editing.

## Declaration of competing interest

The authors declare no conflicts of interest.
